# Combination of Capped Gold Nanoslit Array and Electrochemistry for Sensitive Aqueous Mercuric Ions Detection

**DOI:** 10.3390/nano12010088

**Published:** 2021-12-29

**Authors:** Cheng-Chuan Chen, Shu-Cheng Lo, Pei-Kuen Wei

**Affiliations:** 1 Research Center for Applied Sciences, Academia Sinica, Taipei 11529, Taiwan; ccchen700106@gmail.com (C.-C.C.); bookshawnijz@gmail.com (S.-C.L.); 2 Institute of Applied Mechanics, National Taiwan University, Taipei 11529, Taiwan; 3 Institute of Biophotonics, National Yang-Ming University, Taipei 11221, Taiwan

**Keywords:** gold nanoslits, surface plasmon, electrochemistry, mercury ions

## Abstract

Label-free surface plasmon resonance (SPR) detection of mercuric ions in various aqueous solutions, using capped gold nanoslit arrays combined with electrochemical (EC) sensing technique, is demonstrated. The nanoslit arrays are fabricated on flexible cyclo-olefin polymer substrates by a nanoimprinting lithography method. The EC and SPR signals for the investigation of current responses and transmission SPR spectra are simultaneously measured during metal ions electrodeposition. Glycerol–water solution is studied to evaluate the resonant peak wavelength sensitivity (480.3 nm RIU^−1^) with a FOM of 40.0 RIU^−1^ and the obtained intensity sensitivity is 1819.9%. The ferrocyanide/ferricyanide redox couple performs the diffusion controlled electrochemical processes (*R^2^* = 0.99). By investigating the SPR intensity changes and wavelength shifts of various mercuric ion concentrations, the optical properties are evaluated under chronoamperometric conditions. The sensors are evaluated in the detection range between 100 μM and 10 nM with a detection limit of 1 μM. The time dependence of SPR signals and the selectivity of 10 μM Hg^2+^ in the presence of 10 μM interfering metal ion species from Ca^2+^, Co^2+^, Ni^2+^, Na^+^, Cu^2+^, Pb^2 +^ and Mn^2+^ are determined. The capped gold nanoslit arrays show the selectivity of Hg^2+^ and the EC sensing method is effectively utilized to aqueous Hg^2+^ detection. This study provides a label-free detection technique of mercuric ions and this developed system is potentially applicable to detecting chemicals and biomolecules.

## 1. Introduction

In recent years, the development of highly sensitive detection using label-free assays on chip scale nanosensors has attracted great attention [1,2]. Label-free detection is not required to use a tagging agent, which has affinity to the target ligands [3,4] and is effectively employed to real time monitoring. Depending on the extensive sensing approaches, electroanalysis [5], colorimetric assay [6], optical detection [7], and mechanical characterization [8] have been applied for the sensitive, selective and label-free detection capability. Surface plasmon resonance (SPR), a commercially mature method, is considered as the sensitively optical biosensor and has been used for catalytic reaction [9], and biomolecular interaction analysis [10]. The SPR has a high surface sensitivity to the dielectric materials in contact with the sensing surface with the plasmonic properties [11], and can be used in situ detection of the amount or concentration of analytes. Moreover, it has been realized that SPR can be performed with electrical analysis based on detecting optical signals by applying potential on the metallic film of the SPR sensor [12]. This indicates that electrochemical (EC) analysis combined with optical detection provides valuable information for investigating the physical property, chemical phenomena, reaction mechanism and kinetic parameters in the label-free detection field.

SPR is known as one of the label-free detection methods and widely applied to analyze biomolecular interactions by measuring the change of the surface refractive index [3]. The optical signal from SPR measurement is proportional to the amount of target analyte. For enhancing the sensitivity and selectivity of analyzed molecules, functional capture molecules are modified on gold surfaces (e.g., DNA and molecularly imprinted polymer) [13,14]. The shortcomings are the issues of time-consuming, non-specific response and intensive cost from the chemical modification. In recent years, the combination of EC and surface plasmon resonance (EC-SPR) has become a powerful tool to study the surface chemical reaction process. The process is known as an oxidation–reduction reaction [2,15]. The recent report demonstrated that the optical, dielectric and electrochemical properties on the deposited metal film can be monitored in real time from the EC-SPR detection [16,17]. Among noble metals, gold metal is usually chosen as the plasmonic material because it is chemically inert, biocompatible, easy to modify and adapted for electromagnetic oscillations [18]. The typical setup of EC-SPR is to use an optical prism to join light onto the gold surface. The gold surface also acts as a working electrode. However, the system is usually bulky and needs a precise control of incident angle for exciting the SPR. In this work, we replaced the gold coated prism with capped gold nanoslit array on a plastic substrate. The capped gold nanoslit array has good sensitivity to surface refractive index changes [19,20]. It can be used as a working electrode in the three-electrode EC measurement. The optical setup is quite simple and cost-effective by transmission spectrum measurement. We demonstrated this new EC-SPR system and employed it to detect mercury ions in various aqueous solutions. The detection is based on the electrodeposition of mercury (Hg) on gold (Au) thin film at a fixed potential (−0.4 V). It has been reported that the formation of Hg-Au amalgams were found due to the migration of mercury metal into the gold film [21]. The changes of refractive indices between pure Au and Au-Hg amalgam led the shifts of the resonance signal to a slow potential sweep. This reveals that the combination of EC and SPR analysis is a feasible technique for directly detecting mercuric ions in situ. Herein, the cost-effectiveness and ease of operation of EC-gold nanoslit setup is applied for detecting mercury ions with different interference ions. There are couples of sensing methods recently developed for the detections of mercuric ions, such as quartz crystal microbalance (QCM), fluorescence and spectral methods. These sensing methods have demonstrated good ability to detect mercuric ions, but may require expensive equipment, labelling materials, time-consuming processes or complicated surface modification (we show this in Supplementary Table S1). This work combines EC method and proposes a low-cost, label-free and fast detection method for mercury ions without additional surface modification. However, there has been a lot of work using the electrochemical method for heavy metal detection during recent years [22,23]. For the use of the EC approach alone, the detection limit (LOD) is not good enough. Therefore, these methods all need specific surface modification and additional materials such as nanoparticles to enhance the detection limit. The following table shows the comparison between these works and the additional processes (Supplementary Table S2). Our work proposes a low-cost, label-free and fast detection method for mercury ion detection without additional surface modification and nanoparticle enhancement. The results verify the high sensitivity of capped gold nanoslit arrays and good selectivity of mercury ion detection.

## 2. Materials and Methods

All chemicals used were analytical grade without any further purification and purchased from Sigma Aldrich (Burlington, MA, USA). Mercury chloride (HgCl_2_) and potassium chloride (KCl) were used as the detecting ion and electrolyte. Calcium chloride (CaCl_2_), cobalt chloride (CoCl_2_), nickel chloride (NiCl_2_), copper oxide (CuO) and lead chloride (PbCl_2_) were used as interfering metal ions in this study. Potassium hexacyanoferrate (III) (K_3_Fe(CN)_6_) and potassium hexacyanoferrate (II) trihydrate (K_4_Fe(CN)_6_·3H_2_O) were used as the redox mediators for the study of scan rate dependence of electrochemical properties. Glycerol–water solutions contained different contents of glycerol (0–20%) and were used as the immersion medium with various refractive indices, including 1.3333, 1.3355, 1.3405, 1.3440, 1.3485, 1.3520, 1.3555, 1.3590 and 1.3625 RIU. All aqueous solutions were prepared using double distilled water (18.2 MΩ cm).

### 2.1. Fabrication of Gold Nanoslit Arrays

Nanoslit arrays with a 500 nm period fabricated by the nanoimprinting lithography method were employed as the EC-SPR sensor. The nanoslit arrays were formed on the flexible cyclo-olefin polymer (COP) Zeonerfilm^®^ layers (ZF16-188, Chuo-ku, Tokyo, Japan) by the thermal-annealing-assisted template-stripping method [24]. A 188 µm thick COP substrate was placed on the template coupled with the silicon template and heated at a temperature of 185 °C to soften the COP substrate. Then, the silicon mold and COP substrate were pressed with the vacuum pressing process. The nanoslit arrays structured COP substrate was capped with 50 nm thick gold thin film using DC sputtering. The gold nanoslit arrays were also used as a working electrode for EC-SPR detection. The 500 nm period nanoslits resulted in a transmittance peak at 690 nm wavelength due to the SPR effect. Although the transparent COP plastic substrate was coated with 50 nm thick gold film, the SPR signal is clear and can be easily detected with the spectrometer.

### 2.2. Characterization of the Nanostructures

**Figure 1:**
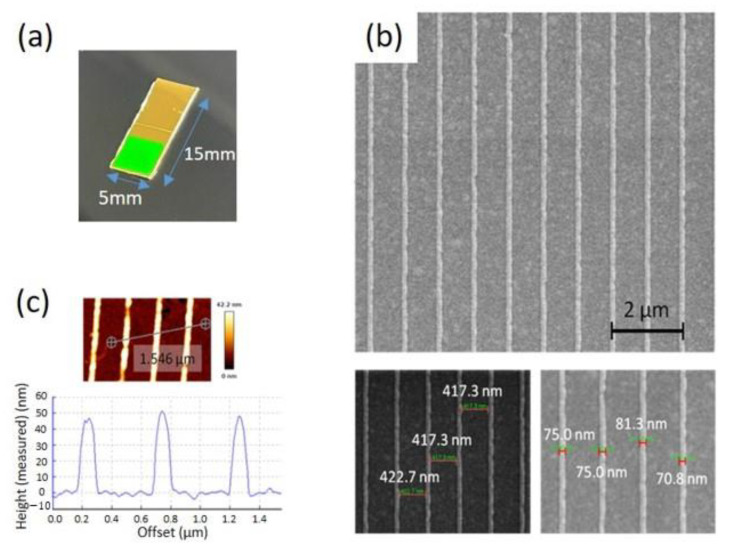
Surface characterization of a COP based capped gold nanoslit arrays: (**a**) the photographic image of EC-SPR sensor; (**b**) SEM images and nanoslit width measurement; (**c**) surface roughness and nanoslit height measurement using atomic force microscopy.

Figure 1a shows the optical picture of a gold capped EC-SPR sensor. The surface morphology of the gold nanoslit arrays was determined using a Helios NanoLab 660 focused ion beam scanning electron microscope (FIB-SEM) (FEI, Hillsboro, OR, USA). The characterized width using SEM for the capped gold nanoslit arrays is about 75.0 nm and the outside surface is about 417.3 nm (Figure 1b). The depth of the gold nanostructures on the COP substrate was analyzed by atomic force microscopy line scan (BioAFM NanoWizard 3, JPK, Berlin, Germany), operated in AC mode under dry conditions and using highly doped silicon to dissipate static charge tips (PPP-RT-NCHR, Nanosensors, Neuchatel, Switzerland) with a spring constant of 42 N/m and a resonant frequency of 330 kHz. Figure 1c shows the cross-sectional profile of template-stripped nanostructures. The measured height of capped gold nanoslit arrays is about 47 nm.

### 2.3. Electrochemical and Optical Setup

**Figure 2:**
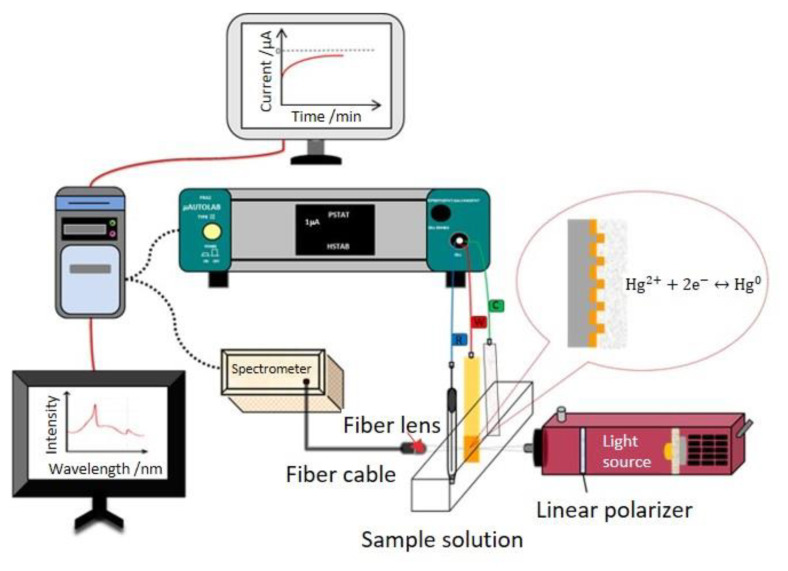
Schematic representation of the EC-SPR system for mercury ion detection.

A standard three-electrode cell, connected to an electrochemical analyzer, was used to characterize the electrochemical behaviors. The capped gold nanoslit arrays-based sensor was used as a working electrode. The reference and counter electrodes were an Ag/AgCl electrode (3 M KCl solution) and a platinum film electrode. The electrochemical measurements were performed using a programmable potentiostat-galvanostat µAutolabIII/FRA2 (Metrohm-Autolab, Utrecht, The Netherlands). The sample solution was injected into a 3 mL plastic cuvette for EC-SPR measurement (Figure 2). The cyclic voltammetry (CV) in the potential range from −0.2 V to +0.8 V at different scan rates of 25, 50, 100, 200, 400 and 800 mV _S_^−1^ in 10 mM K_3_Fe(CN)_6_ and 10 mM K_4_Fe(CN)_6_·3H_2_O mixture containing 10 mM KCl solution [25,26]. To obtain an optimal applied potential in this EC-SPR system, the chronoamperometry (CA) measurements for studying the changes of optical properties in 100 μM HgCl_2_ containing 10 mM KCl were performed at various potentials of −0.1, −0.2, −0.3, −0.4, −0.5 and −0.6 V for 25 min. The detection range of capped gold nanoslit arrays-based EC-SPR sensors were investigated in various HgCl_2_ concentrations containing 10 mM KCl. To study the selectivity, the variations of SPR properties for Hg^2+^ and interfering metal ions mixed solutions were performed under a −0.4 V applied potential. All electrochemical measurements were performed under the ambient environment. The current signals received from µAutolabIII/FRA2 were analyzed by NOVA 1.11 (Metrohm Autolab, Utrecht, The Netherlands).

The transmission SPR spectra were measured by a simple optical setup (Figure 2). A stabilized tungsten halogen light source with a 9 W bulb power was used as a light source and the linear polarizer was placed between the lamp and sample solution. A polarized light beam was focused by a 10× objective lens. After collimated light passed through the sample, the transmission light was collected by a fiber lens with a fiber cable. All transmission spectra were acquired by a CCD-based spectrometer with a spectral resolution of 0.3 nm (B&W Tek Inc., Newark, DE, USA). Time-dependent data of optical analysis saved from the spectrometer were processed and analyzed by the developed algorithm using MATLAB (The MathWorks Inc., Natick, MA, USA).

## 3. Results and Discussion

### 3.1. Optical and Electrochemical Properties on Capped Gold Nanoslit Arrays

**Figure 3:**
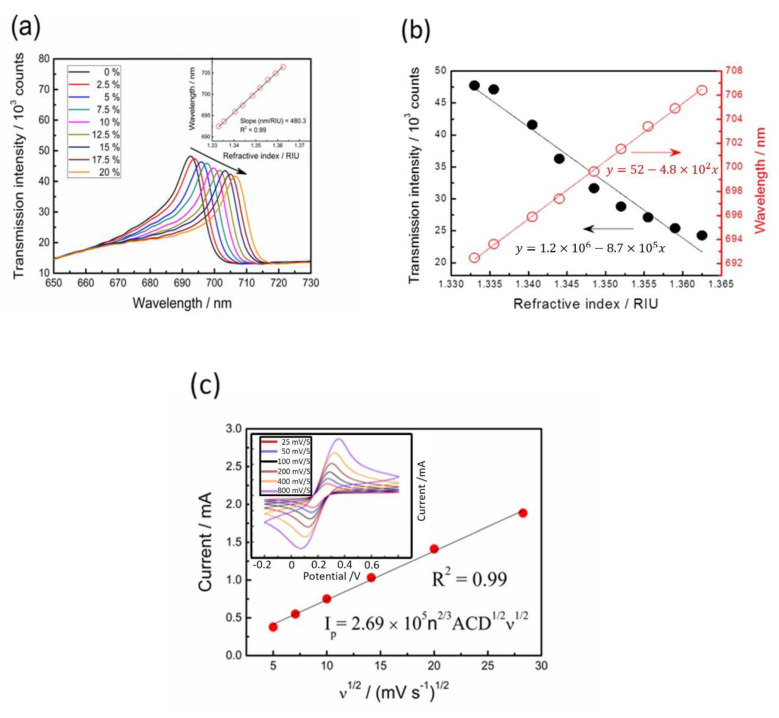
Characterization of the optical properties and electrochemical behaviors on EC-SPR sensors: (**a**) transmission spectra of capped gold nanoslit arrays in glycerol–water solutions with different environmental refractive indices; (**b**) the intensity and wavelength changes as a function of refractive index; (**c**) the relationship between the square root of scan rate and peak current from cyclic voltammograms.

The wavelength and intensity sensitivities were evaluated using the glycerol–water solutions with different refractive indices (*n*) between 1.3333 and 1.3625. The resonant peak wavelength (*λ*) and intensity (*I*) obtained from the transmission spectra were both recorded when the capped gold nanoslit arrays-based sensor was immersed in the glycerol–water solutions. The transmission intensities of the resonance peaks show a continued decrease with the longer wavelength shifts (red shifts) when the refractive index increased (Figure 3a). The results of the refractive indices for different glycerol–water solutions and the resonant peak transmission show intensities and wavelengths (Figure 3b). The main reason for detecting different concentrations of glycerol is to demonstrate the sensing performance of SPR. The SPR sensor is sensitive on the surrounding medium due to the distribution of electrical field on the interface. Different concentrations of glycerol could be simulated by the different refractive index unit of the bulk solution. The wavelength (*S_λ_*) sensitivities and transmission intensity (*S_I_*) to the refractive index changes are represented as follows:

(1) $$S_{\lambda }=\frac{\partial \lambda }{\partial n}$$

(2) $$S_{I}=\left(\frac{\partial I}{\partial n}\right)/I_{0}\times 100\%$$

where *I*_0_ is the referenced intensity. In the measurement, the transmission intensity in water (*n* = 1.3333) was set as *I*_0_. The wavelength sensitivity of the capped gold nanoslit arrays (the linear regression slope for the refractive index) was calculated from the resonance wavelength change (Equation (1)) and was estimated as 480.3 nm RIU^−1^. Furthermore, the intensity sensitivity of 1819.9% at 693.2 nm is obtained from the relative intensity change over the refractive index change at a fixed wavelength near resonance (Equation (2)). The figure of merit (FOM) defined as the quotient of wavelength sensitivity and full width at half maximum is an important parameter for the quality evaluation of a sensor. In this study, the corresponding FOM value was calculated from the resonance peaks as 40.0 RIU^−1^.

Cyclic voltammetry is an extensively used electrochemical technique for the investigation of chemical reactions at the interface between liquid and electrode surface. In this study, the electrochemical behaviors depending on a redox pair of K_3_Fe(CN)_6_/K_4_Fe(CN)_6_ were performed for the characterization of capped gold nanoslit arrays-based working electrode at different scan rates of 25, 50, 100, 200, 400 and 800 mV _S_^−1^ between −0.2 V and +0.8 V. The responding currents as a function of the applied potentials are plotted (inset of Figure 3c). It can be noted that the redox reaction is electrochemically reversible because both oxidation and reduction peaks are identical. The anodic peak currents in the forward scans increase with the square root of scan rates (Figure 3c). According to the Randles–Sevcik equation (Equation (3)), the redox reaction between K_3_Fe(CN)_6_/K_4_Fe(CN)_6_ on capped gold nanoslit arrays display a typical diffusion controlled process [27].

(3) $$I_{P}=2.68\times 10^{5}n^{2/3}ACD^{1/2}v^{1/2}$$

where *I_p_* = peak current (A); *n* = electron stoichiometry of electrons passed (e.g., *n* = 1 for Fe(CN)_6_^3−^ + e^−^ ↔ Fe(CN)_6_^4−^); *A* = reaction area (cm^2^), and detection area of SPR electrode sensor; *C* = concentration (mol cm^−3^); *D* = diffusion coefficient (cm^2^ s^−1^); *ν*= scan rate (V s^−1^). The *n*^2/3^*ACD*^1/2^ could be considered as constant in this system and is approximately 2.6 × 10^−7^ ± 0.1 × 10^−7^ mA s V^−1^. Hence, it can be observed that the developed EC-SPR sensor has optical and electrochemical capabilities for the investigation of electrochemical reactions of metal ions.

### 3.2. EC-SPR Analysis for Hg^2+^ Detection on Gold Nanoslit Arrays

**Figure 4:**
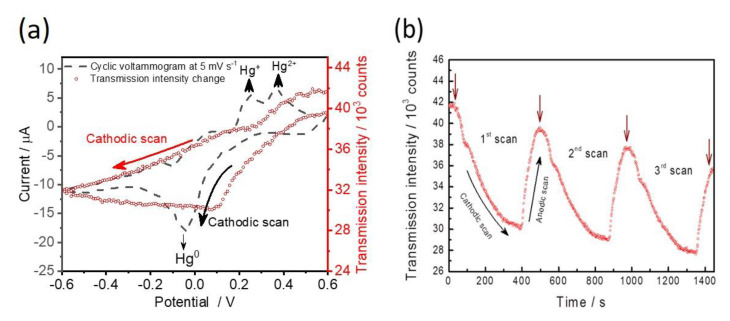
(**a**) Redox current curves during the first cyclic voltammetry scan and the simultaneously measured SPR intensity. The filled squares indicate current and the open diamonds indicate SPR intensity. (**b**) The changes of SPR intensity during three cyclic voltammetry scans. Brown arrows point out the transmission intensities at +0.6 V for each cycle. Electrolyte: 100 μM Hg^2+^ in 10 mM KCl solution. Scan rate: 5 mV s^−1^.

For the investigation of EC-SPR performances and heavy metal ion sensing application, capped gold nanoslit arrays were employed to detect Hg^2+^ concentrations. Electrodeposition and electro-reoxidation of Hg^2+^ are accompanied using cyclic voltammetry scans with a lower scan rate of 5 mV s^−1^ at a potential range between −0.6 V and +0.6 V [21]. The transmission intensities and generated electrochemical currents were recorded simultaneously at various applied potentials. The CV voltammograms and the transmission intensity changes of 100 μM Hg^2+^ are measured during the potential sweep (Figure 4a). The linear sweep potential started at +0.6 V with a cathodic scan that drives the electrodeposition of Hg^2+^ on the gold surface as Equation (4).

(4) $${\mathrm{Hg}}^{2+}+2e^{-}\Leftrightarrow {\mathrm{Hg}}^{0}$$

There is a strong cathodic peak current, which occurred at −0.02 V during the cathodic scan. It is seen that the decrease of transmission intensity depends on the decrease of potential from +0.6 V until −0.6 V during the cathodic scan. The electrochemical Hg^2+^ reduction current peak from the formation of metallic Hg^0^ was observed from +0.2 V and −0.2 V (Figure 4a). It is known that the deposition thickness of Hg^0^ is proportional to the SPR signal change. In comparison between the current signal response and the SPR signal change, the change of transmission intensity between +0.6 V and +0.2 V is more obvious than that of the electrochemical current. The result indicates that the optical property measurement is more sensitive than the electrochemical analysis for the detection of Hg^2+^. During the anodic scan, the deposited Hg^0^ metal was oxidized to Hg^2+^ ion and generated oxidizing currents. According to the transmission intensity change from −0.6 V to +0.6 V, the SPR intensity slightly decreased from −0.6 V to +0.08 V. When the applied potential was higher than +0.1 V, the oxidation process of Hg^0^ substantially increased the SPR intensity and reached close to the initial state at +0.6 V. As a result of the current response, there are two split oxidation peaks depicted at +0.25 V and +0.32 V in the cyclic voltammogram (Figure 4a). It has been reported that the first peak at +0.25 V is assigned as the formation of Hg^+^ and the second peak at +0.32 V corresponds to the formation of Hg^2+^ [17,21]. The successive CV sweeps with three cycles from the start potential of +0.6 V to the lowest potential of −0.6 V were studied (Figure 4b). The highest resonance transmission intensities gradually decrease with potential sweep times ranging from 0 to 25 min. This indicates that the electrochemical reaction between the electrodeposition of Hg^2+^ and the re-oxidation of Hg^0^ is partially irreversible on gold nanoslit arrays.

**Figure 5:**
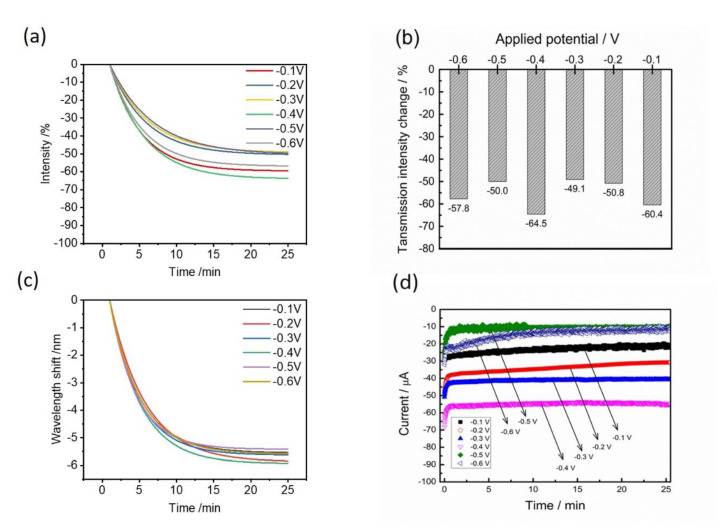
(**a**) Time-dependent SPR intensity changes and (**b**) time-dependent wavelength shifts under various applied potentials. (**c**) The comparison of the steady-state SPR intensity and wavelength changes under various applied potentials. (**d**) The electrochemical current responses at various applied potentials between −0.1 and −0.6 V. Electrolyte: 100 μM Hg^2+^ in 10 mM KCl solution.

From a practical aspect, the chronoamperometry method with a constant applied potential is more achievable than the cyclic voltammetry method. The CV method usually needs an accurate and high resolution of the equipment, but the chronoamperometry method has a much lower cost and is easily set up by a DC voltage. Moreover, the detection sensitivity of the EC-SPR method for Hg^2+^ detection is determined by the applied potential and deposition times. Hence, the optical performances of transmission changes and wavelength shifts were characterized under various applied potentials from −0.1 V to −0.6 V (Figure 5). According to the results, the decreases of the transmission intensities at 20 min are −60.4, −50.8, −49.1, −64.5, −50.0 and −57.8% for various applied potentials of −0.1, −0.2, −0.3, −0.4, −0.5 and −0.6 V (Figure 5a). The resulting wavelength shifts rapidly reach the equivalent values within 20 min but −0.4 V owns the largest wavelength shift and is distinguishable in the potential ranges (Figure 5b). Figure 5c shows the SPR signal changes at 25 min reaction time for various applied voltages. It can be seen that −0.4 V has a more appropriate applied potential for further studies due to the highest change of the intensity change of −64.5% and wavelength shift (−6 nm). According to the influence of the applied potential, the electrochemical currents were simultaneously obtained at various applied potentials (Figure 5d). The highest reduction current (−58 μA) from Hg^2+^ electrodeposition also occurred at about −0.4 V.

### 3.3. Sensing Performance Evaluation for Hg^2+^ Determination

**Figure 6:**
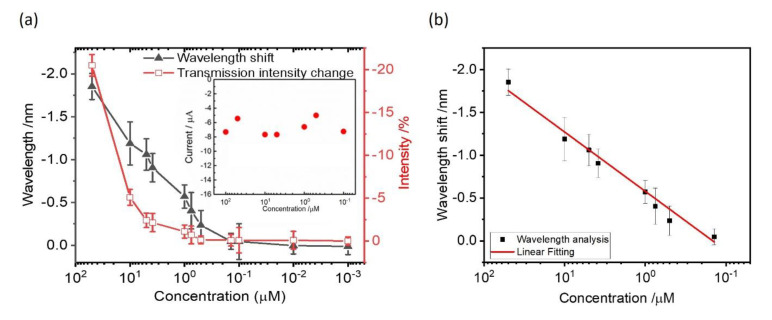
(**a**)The wavelength shifts and the transmission intensity changes of gold nanoslit arrays against Hg^2+^ concentrations between 100 μM and 1 nM. The inset shows the reduction reaction currents of amperometric responses at various Hg^2+^ concentrations. (**b**) The measured wavelength shifts against the logarithm of the Hg^2+^ concentration. The measured points are fitted with a red line in the semi-log plot. The fitting equation is ΔSPR signal (nm) = −0.69 log (C) − 4.7 with a correlation coefficient of *R*^2^ = 0.98, where C is the molar concentration (M) of Hg ions.

The detection range of Hg^2+^ on gold nanoslit arrays using chronoamperometry was evaluated by the SPR signals with the Hg^2+^ range between 100 μM and 1 nM. From the corresponding curves in the preliminary study, the obtained resonant peak signals of 1 mM and 500 μM change quickly and the transmission intensities reached the steady states within 10 min. The optical photography of the chronoamperometric deposited Hg^2+^ on gold nanoslit arrays at 1 mM and the silvery–white film with a mirror-like appearance corresponds to Hg^0^ metal. This is because the fast electrodeposition of Hg^2+^ at such high concentrations leads to a deposit of thick Hg^0^ metallic film on gold surfaces. Hence, the maximum concentration of Hg^2+^ for the study of sensitivity and dynamic range was limited at 100 μM as the proper condition. The transmission intensity changes and wavelength shifts through the reduction potential of −0.4 V with various Hg^2+^ concentrations were investigated. The transmission intensity changes and the wavelength shifts increased with the increasing concentration of Hg^2+^. We repeated the experiment using different SPR chips and measured at a different time. Figure 6a shows the SPR wavelength shifts, intensity changes and the corresponding error bars as a function of the logarithm of the Hg^2+^ concentration. The SPR signal can be extracted from the wavelength or intensity changes. It is well known that SPR signals are linearly changed with the logarithmic concentration of the analyte. The wavelength has a large dynamic measurement range. However, the SPR intensity signal has a small dynamic range. In the measurement, the intensity signals become nonlinear for higher concentrations. Figure 6b shows the measured wavelength shifts against the logarithm of the Hg^2+^ concentration. A linear relationship was found within the concentration range from 0.1 μM to 100 μM. The linear fitting is ΔSPR signal (nm) = −0.69 log (C) − 4.7 with a correlation coefficient of *R*^2^ = 0.98, where C is the molar concentration (M) of Hg ions. The limit of quantification (LOQ) and the limit of detection (LOD) as estimated from experimental results are about 2.5 μM and 1 μM, respectively. The LOD was determined by interpolating the mean SPR response obtained for the blank of five replicates plus three times its standard deviation in the linear equation. The LOD could be further improved through integration with microfluidic devices [3]. In comparison to Hg^2+^ detection sensitivities between optical and electrochemical sensing techniques, the reduction reaction currents of amperometric responses at various Hg^2+^ concentrations at 20 min were recorded (inset of Figure 5a). The generated reduction currents are −7.3, −5.4, −7.7, −7.7, −6.6, −5.0 and −7.2 μA for 100 μM, 50 μM, 10 μM, 5 μM, 1 μM, 500 nM and 100 nM Hg^2+^ containing 10 mM KCl, respectively. It can be seen that the current signals from a fixed applied potential of −0.4 V are not distinguishable from various Hg^2+^ concentrations with the background current of −6.9 μA. The background current came from 10 mM KCl in the buffer solution. Compared to the EC current, the SPR signals from the capped gold nanoslit electrode show significant wavelength shifts and intensity changes. It verifies the sensitive aqueous mercuric ions detection using capped gold nanoslit array and electrochemistry. We also measured and compared the real sample using different samples, including deionized water, drinking water and tap water. With the exception of H_3_O^+^ and OH^−^, deionized water does not contain any other ionic components. Drinking water was obtained from a commercial water dispenser which has semi-permeable membrane to filter heavy metals, pesticides, viruses and other particles from tap water. Different concentrations of mercury dichloride were added in these water samples. However, as foreign substrates increase (tap water > drinking water > deionized water) in the sample, the signals were reduced (we show these in Supplementary Figure S4).

### 3.4. Determination of Hg^2+^ in Different Interfering Ion Solutions

**Figure 7:**
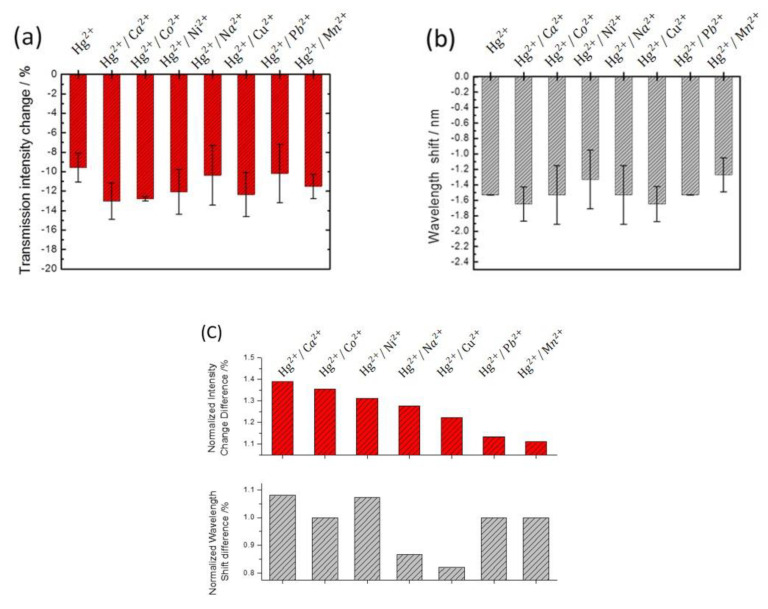
The selectivity of EC-SPR sensors by investigating (**a**) the transmission intensity changes and (**b**) the wavelength shifts for 10 μM Hg^2+^ in the presence of 10 μM interfering ions in 10 mM KCl solution, including Ca^2+^, Co^2+^, Ni^2+^, Na^+^, Cu^2+^, Pb^2+^ and Mn^2+^. (**c**) The variations of the normalized transmission intensity changes and the wavelength shifts of mixing ions compared to Hg^2+^ at 20 min reaction time. The error bars represent the standard deviation from three repeated tests.

The capped gold nanoslit arrays-based sensors were used to examine the selectivity of Hg^2+^ in aqueous solution using mixed ion solutions with various interfering ions to simulate the industrial wastewater or some agriculture environment. The interfering metal ion solutions, including Ca^2+^, Co^2+^, Ni^2+^, Na^+^, Cu^2+^, Pb^2+^ and Mn^2+^ ions, were investigated in the presence of Hg^2+^ ions (Figure 7). It can be seen that the transmission intensities decrease in 10 μM interfering metal ion solutions. This is because the interfering ions are electroactive species which formed the reduced metal on gold surfaces during the chronoamperometric process. However, the interfering ions were found not to significantly affect the wavelength shift on gold nanoslit arrays except Cu^2+^ and Pb^2+^ due to the variation of dielectric constant values for the interfering ions. The results indicated that Hg^2+^/Ca^2+^ and Hg^2+^/Cu^2+^ solutions showed the relatively blue-shifts of −0.12 and −0.12 nm and Hg^2+^/Ni^2+^ and Hg^2+^/Mn^2+^ solutions performed the relatively red-shifts of +0.20 and +0.26 nm compared to the Hg^2+^ solution. Moreover, there is no significant change of SPR peak wavelength in the Hg^2+^/Co^2+^, Hg^2+^/Na^+^ and Hg^2+^/Pb^2+^ solutions. The variation of wavelength shift was attributed to the selectivity of the chronoamperometric method depending on the difference of standard reduction potentials for the interfering ions and the change in dielectric constant for the reduced metal on gold surfaces. To analyze the variation of the optical performances in the mixed ion solutions, the differentials of the normalized wavelength shifts and transmission intensities were compared, as seen in Figure 7c. The variations of wavelength shifts are −0.12, 0, +0.20, 0, −0.12, 0 and +0.26 nm for 10 μM Hg^2+^ solutions in the presence of 10 μM Ca^2+^, Co^2+^, Ni^2+^, Na^+^, Cu^2+^, Pb^2+^ and Mn^2+^ ions, respectively. According to the results of the wavelength shifts, the proposed capped gold nanoslit arrays-based sensors performed the Hg^2+^ ion selectivity of 10 μM concentration in the mixed ion solutions and were favorable for the label-free detection of Hg^2+^ in aqueous solution. The EC-SPR based on the wavelength shifts can also be used to detect other heavy metal ions [28,29,30]. The potential performance is different of these ions, which could help experiments to know the qualitative ions and propose an analyzed method by using SPR in aqueous solutions.

## 4. Conclusions

This study aims to demonstrate a label-free technique for Hg^2+^ ion detection using capped gold nanoslit arrays-based sensors, combined with the electrochemical surface plasmon resonance method. The three-electrode electrochemical analysis and optical transmission measurement was employed to characterize the potential current responses and the resonant peak signals for the investigation of metal ion electrodeposition. The nanostructured EC-SPR sensors were used to characterize the electrochemical behaviors of K_3_Fe(CN)_6_/K_4_Fe(CN)_6_ redox couple and evaluate the wavelength sensitivity (480.3 nm RIU^−1^) with an FOM of 40.0 RIU^−1^ and the intensity sensitivity (1819.9%) in the glycerol–water solutions. The detection limit of 1 μM Hg^2+^ can be obtained by the chronoamperometric-spectrum analysis. The developed capped gold nanoslit arrays-based sensors present Hg^2+^ ion selectivity over the wavelength shifts of the interfering ions including Ca^2+^, Co^2+^, Ni^2+^, Na^+^, Cu^2+^, Pb^2+^ and Mn^2+^ ions. It significantly demonstrated the capacity of capped gold nanoslit as a rapid, label-free, sensitive and high ion-selective EC-SPR platform for aqueous Hg^2+^ detection.

## Data Availability

The data is available on reasonable request from the corresponding author.

## Supplementary Materials

The following supporting information can be downloaded at: https://www.mdpi.com/article/10.3390/nano12010088/s1. Figure S1: Tested nine samples and show its spectrum for the reproduction of nanoslit array; Figure S2: Tested 20 samples and show mean SPR peak wavelength and full width at half maximum (FWHM) of the SPR; Figure S3: The durability and stability of the nanoslit array in the medium for 2 h; Figure S4: The result of using different samples, including deionized water, drinking water, and tap water. The measured peak wavelength shifts for different samples as a function of mercury ion concentrations; Table S1: Some significant and commercial sensing technology of mercuric ions. Comparison of limit of detection (LOD), real sample and disadvantages; Table S2: Some significant EC technology of detecting mercuric ions. Comparison of limit of detection, real sample and additional process.
