# Supplementary material for: Combination of Capped Gold Nanoslit Array and Electrochemistry for Sensitive Aqueous Mercuric Ions Detection
Source: Nanomaterials (Basel). 2021 Dec 29;12(1):88. doi: 10.3390/nano12010088 (PMC8746490; doi:10.3390/nano12010088)
Supplement: Supplementary file 1 [file nanomaterials-12-00088-s001.zip › nanomaterials-1486916-supplementary.pdf]

nanomaterials

Supplementary Information

Combination of Capped Gold Nanoslit Array and Electrochemistry for Sensitive Aqueous Mercuric Ions Detection

Cheng-Chuan Chen [1,†], Shu-Cheng Lo [1,2,†] and Pei-Kuen Wei [1,3,*]

1 Research Center for Applied Sciences, Academia Sinica, Taipei 11529, Taiwan; ccchen700106@gmail.com (C.-C.C.); bookshawnijz@gmail.com (S.-C.L.)
2 Institute of Applied Mechanics, National Taiwan University, Taipei 11529, Taiwan
3 Institute of Biophotonics, National Yang-Ming University, Taipei 11221, Taiwan
* Correspondence: pkwei@sinica.edu.tw
† These authors contributed equally to this work.

1. There are couples of sensing methods recently developed for the detections of mercuric ions, such as quartz crystal microbalance (QCM), fluorescence and spectral methods. [1] These sensing methods have demonstrated good ability to detect mercuric ions, but may require expensive equipment, labelling materials, time-consuming process or complicated surface modification. This work proposes a low-cost, label-free, and fast detection method for mercury ions without additional surface modification.

Table S1. Some significant and commercial sensing technology of mercuric ions. Comparison of limit of detection (LOD), real sample and disadvantages.

Technology	LOD (μM)	Sample	Disadvantage	Ref
Quartz crystal microbalance	5	Deionized water	Covalent organic framework needed	[2]
fluorescence	0.63	Tap water	Label	[3]
Mass spectrometry	2.2×10^{-4}	Surface water	Expensive equipment	[4]
Raman spectrometry	8.3×10^{-3}	Dry	Pure sample, expensive equipment	[5]
Absorbance	5×10^{-2}	Sewage water	Functional Ag NPs needed	[6]
X-ray Spectrometer	5.2×10^{-3}	Sea water	Expensive equipment	[7]

2. Reproducibility between different nanoslit array.

For the reproduction of nanoslit array, we have tested nine samples as shown in the following Figure S1. The nanoslit arrays on plastic substrates were fabricated by the nanoimprinting lithography. The nanoslit-structured substrates were coated with 50-nm thick gold film using DC sputtering. The mean SPR peak wavelength of the 20 samples is about 670.3nm and the standard deviation is only 0.93nm. The mean full width at half maximum (FWHM) of the SPR is about 18.5nm with the standard deviation of 1.5 nm. The SPR spectra for nine nanoslit arrays and the mean and variation values are shown in Figure S2.

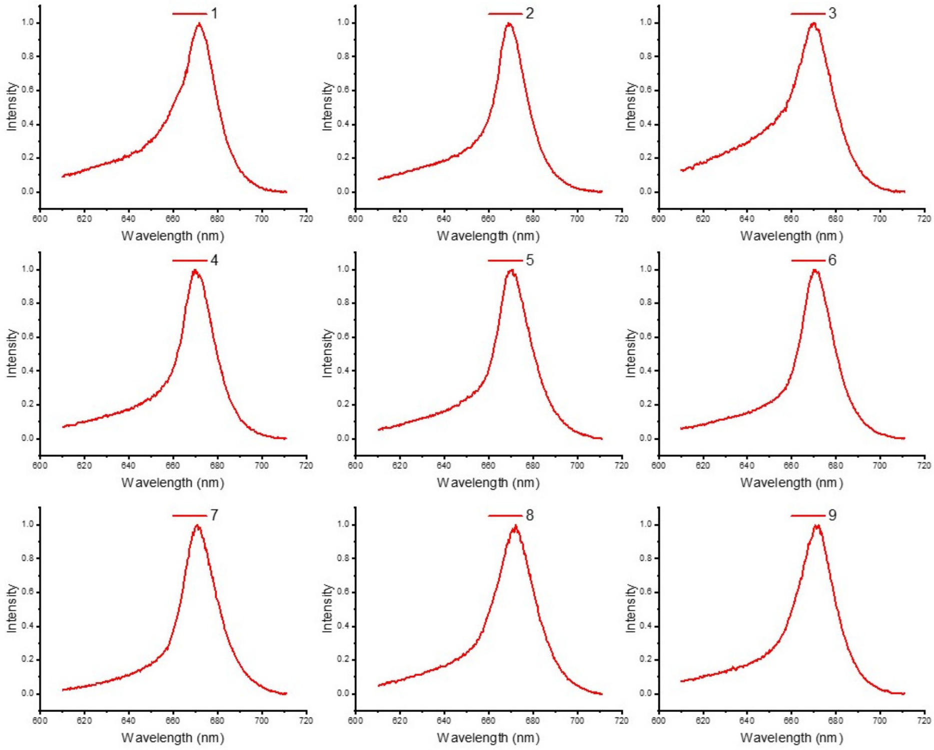

Figure S1. Tested nine samples and show its spectrum for the reproduction of nanoslit array.

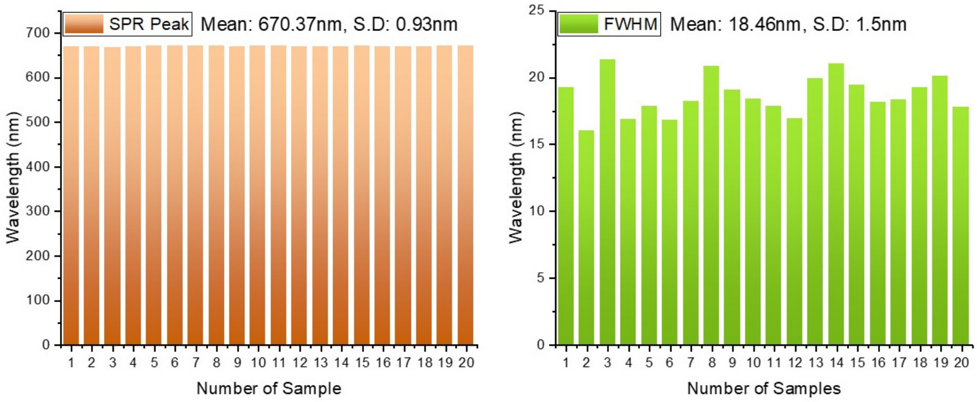

Figure S2. Tested 20 samples and show mean SPR peak wavelength and full width at half maximum (FWHM) of the SPR.

3. The durability and stability of the nanoslit array

To test the durability and stability, we have put nanoslit array in the medium for 2 hours, and the SPR spectra were recorded every 30 seconds. From the result as shown in the following Figure S3. The SPR signals are extremely stable. The intensity noise for the peak wavelength intensity is only about 0.14%. In this work, the mercury ions are detected within 30 minutes. The two-hours test verifies the excellent durability and stability of the nanoslit array and the measurement system.

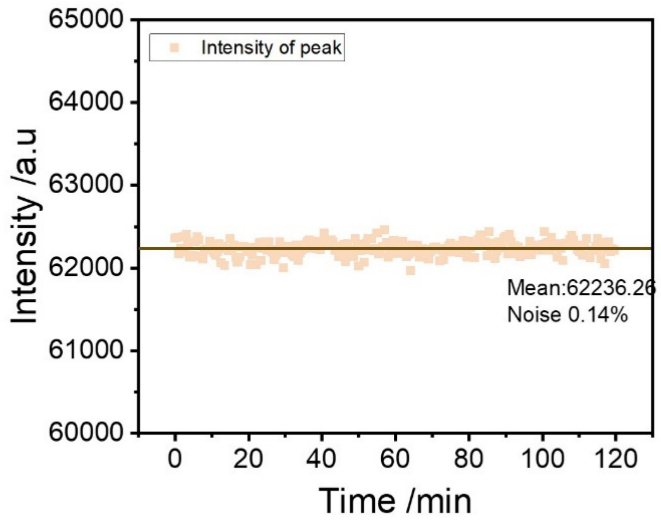

Figure S3. The durability and stability of the nanoslit array in the medium for 2 hours.

4. Testing real sample of water

We added the measurement results using different samples, including deionized water, drinking water, and tap water. Except for H3O+ and OH−, deionized water does not contain any other ionic components. Drinking water is obtained from commercial water dispenser which has semi-permeable membrane to filter heavy metals, pesticides, viruses and other particles from tap water. Different concentrations of mercury dichloride were added in these water samples. The following Figure S4 shows the measured peak wavelength shifts for different samples as a function of mercury ion concentrations. The signals all show significant increase with the increase the mercury ions. However, as foreign substrates increase (tap water > drinking water > deionized water) in the sample, the signals are reduced. For the real application, a calibration curve for the real environment water is necessary.

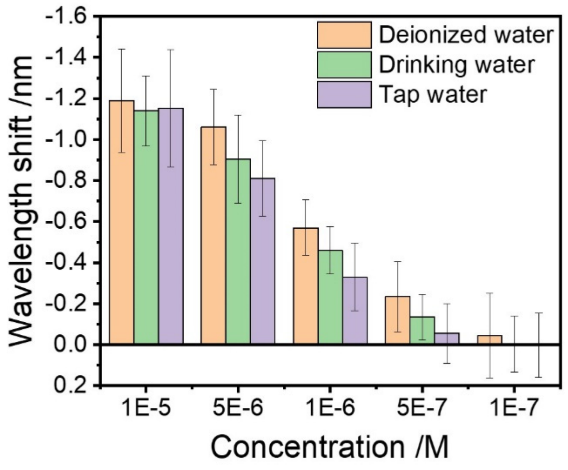

Figure S4. The result of using different samples, including deionized water, drinking water, and tap water. The measured peak wavelength shifts for different samples as a function of mercury ion concentrations.

4. Some references of electrochemical method of Hg ions in water

There are lots of works using electrochemical method for heavy metal detections during recent years [8, 9]. For only using the EC approach, the detection limit (LOD) is not good enough. Therefore, these methods all need specific surface modification and additional materials such as nanoparticles to enhance the detection limit. The following table S2 shows the comparison between these works and the additional processes. Our work proposes a low-cost, label-free, and fast detection method for mercury ions detection without additional surface modification and nanoparticles enhancement.

Table S2. Some significant EC technology of detecting mercuric ions. Comparison of limit of detection, real sample and additional process.

EC-Method	LOD (μM)	Water	Additional Process	Ref
SWV	$5x10^{-4}$	Tap, river water	MSO, linker probes + AuNPs	[10]
DPV	$5x10^{-4}$	DI-water sample	DNA, methylene blue + AuNPs	[11]
DPV	$7.38x10^{-6}$	Tap water	Thiolated probe DNA+ AuNPs	[12]
CV	$1x10^{-2}$	DI-water	MSO, ss-DNA + AuNPs	[13]
DPV	$5x10^{-6}$	Tap water	aptamer/(AuNPs/CS)2/GCE + AuNPs	[14]
SWV	$1x10^{-2}$	Lake water	DNA modified Fe_3O_4/AuNPs	[15]
EC-SPR	<1	Drink /Tap water	None	This work

Abbreviations: Au gold, nanoparticles NPs, SWV Square wave voltammetry, Differential pulse voltammetry DPV.

References

1. Zaib, M., et al., Electrochemical determination of inorganic mercury and arsenic—a review. Biosensors and Bioelectronics, 2015. 74: p. 895-908.
2. Li, D.-M., et al., A recyclable bipyridine-containing covalent organic framework-based QCM sensor for detection of Hg (II) ion in aqueous solution. Journal of Solid State Chemistry, 2021. 302: p. 122421.
3. Yang, Y., et al., Ratiometric fluorescence detection of mercuric ions by sole intrinsic dual-emitting gold nanoclusters. Sensors and Actuators B: Chemical, 2019. 278: p. 82-87.
4. Li, J., et al., Ultrasensitive speciation analysis of mercury in waters by headspace solid-phase microextraction coupled with gas chromatography-triple quadrupole mass spectrometry. Microchemical Journal, 2020. 153: p. 104459.
5. Zhang, X., et al., Ultrasensitive SERS substrate integrated with uniform subnanometer scale “hot spots” created by a graphene spacer for the detection of mercury ions. Small, 2017. 13(9): p. 1603347.
6. Balasurya, S., et al., Colorimetric detection of mercury ions from environmental water sample by using 3-(Trimethoxysilyl) propyl methacrylate functionalized Ag NPs-tryptophan nanoconjugate. Journal of Photochemistry and Photobiology B: Biology, 2020. 207: p. 111888.
7. Koulouridakis, P.E., N.G. Kallithrakas-Kontos, and V.C. Gekas, Seawater mercury analysis at ppb levels. Instrumentation Science and Technology, 2006. 34(4): p. 425-433.
8. Sawan, S., et al., Metal and metal oxide nanoparticles in the voltammetric detection of heavy metals: A review. TrAC Trends in Analytical Chemistry, 2020: p. 116014.
9. Gong, Z., et al., Application of Nanotechnology in Analysis and Removal of Heavy Metals in Food and Water Resources. Nanomaterials, 2021. 11(7): p. 1792.
10. Zhu, Z., et al., Highly sensitive electrochemical sensor for mercury (II) ions by using a mercury-specific oligonucleotide probe and gold nanoparticle-based amplification. Analytical chemistry, 2009. 81(18): p. 7660-7666.
11. Kong, R.-M., et al., An ultrasensitive electrochemical “turn-on” label-free biosensor for Hg2+ with AuNP-functionalized reporter DNA as a signal amplifier. Chemical communications, 2009(37): p. 5633-5635.
12. Tang, X., et al., A fishnet electrochemical Hg 2+ sensing strategy based on gold nanoparticle-bioconjugate and thymine–Hg 2+–thymine coordination chemistry. Analyst, 2012. 137(2): p. 309-311.
13. Miao, P., et al., A novel electrochemical method to detect mercury (II) ions. Electrochemistry Communications, 2009. 11(10): p. 1904-1907.
14. Liu, Y., et al., Aptamer-based electrochemical biosensor for mercury ions detection using AuNPs-modified glass carbon electrode. Journal of biomedical nanotechnology, 2018. 14(12): p. 2156-2161.
15. Miao, P., Y. Tang, and L. Wang, DNA modified Fe3O4@ Au magnetic nanoparticles as selective probes for simultaneous detection of heavy metal ions. ACS applied materials & interfaces, 2017. 9(4): p. 3940-3947.
